# Prevalence and disparities in receiving medical advice to quit tobacco use in the US adult population

**DOI:** 10.3389/fpubh.2024.1383060

**Published:** 2024-09-18

**Authors:** Monalisa Chandra, Rajesh Talluri, Joel Fokom Domgue, Sanjay Shete

**Affiliations:** ^1^Department of Epidemiology, The University of Texas MD Anderson Cancer Center, Houston, TX, United States; ^2^Division of Cancer Prevention and Population Sciences, The University of Texas MD Anderson Cancer Center, Houston, TX, United States; ^3^Department of Biostatistics, The University of Texas MD Anderson Cancer Center, Houston, TX, United States

**Keywords:** medical advice, tobacco cessation, tobacco use, disparities, health care professionals (HCPs)

## Abstract

**Introduction:**

Evidence suggests that advice from health care professionals (HCP) increases the likelihood of quit attempts and successful quitting of tobacco use. However, previous studies primarily focussed on cigarette smoking and did not include all forms of tobacco products. This study aimed to investigate the prevalence and disparities in receiving HCP’s advice to quit tobacco use (combustible or noncombustible) in the US adult population.

**Methods:**

Using the 2022 National Health Information Survey (NHIS) data, we examined 4,424 adults who reported (i) any tobacco product use within the past 12 months and (ii) having seen an HCP within the past 12 months. The outcome variable included the receipt of advice to quit tobacco use from an HCP, and predictors included sociodemographic variables. Weighted prevalence estimates were calculated, and multivariable regression analyses were conducted.

**Results:**

Over 38% of tobacco users who visited an HCP were advised to quit. The odds of receiving such advice were lower among Hispanics (AOR: 0.625; 95% confidence interval (CI) [0.464–0.843];*p* = 0.002), males (AOR: 0.767; 95% CI [0.659–0.893], *p* = 0.001), those above the poverty level (AOR: 0.795; 95% CI [0.641–0.987];*p* = 0.037), foreign-born (AOR: 0.664; 95% CI [0.496–0.888]; *p* = 0.006), those with a bachelor’s degree or higher educational level (AOR: 0.477; 95% CI [0.349–0.653]; *p* < 0.001) and those aged less than 45 years (AOR: 0.404; 95% CI: [0.344–0.473]; *p* < 0.001).

**Conclusion:**

The prevalence of receiving HCP’s advice to quit tobacco use remains suboptimal and disparate among sociodemographic groups. Our findings call for strategic implementation of the USPHS’s recommendation on treating tobacco use and taking further actions to equip HCPs with the training and resources needed to provide appropriate advice to quit tobacco.

## 1 Introduction

Tobacco use is a significant risk factor for several health conditions, such as cancer, cardiovascular disorders, chronic obstructive pulmonary diseases, and oral diseases (1–3). Commercial tobacco can be smoked as cigarettes, e-cigarettes, cigars, cigarillos, little filtered cigars, pipes, hookahs, etc.; consumed smokeless as snuff, loose leaf chewing tobacco, lozenges, etc. (4); or can be used in combination (poly use) (5–7). Despite its public health impact, tobacco use remains a serious health concern. In 2021, it was estimated that nearly 1 in 5 US adults (18.7%) were using any commercial tobacco product (5). Although the prevalence of cigarette smoking (which has been the primary target of most smoking cessation interventions so far) has decreased in the last decade in the US [from 18.0% in 2012 (8) to 11.5% in 2021 (5)], the use of other tobacco products (electronic cigarettes, cigars, cigarillos, little filtered cigars, pipes, and smokeless tobacco) has increased [from 7.3% in 2012 (8) to 11.0% in 2021 (5)].

In 2020, over half (53.9%) of US adults who smoked attempted to quit smoking, and only 8.5% were successful (9). Evidence suggests that advice from healthcare professionals (HCPs) increased the likelihood of quit attempts and successful quitting of tobacco use. Kastaun and colleagues found that patients who received advice from HCPs to quit smoking had 2-fold higher odds of quit attempts and also reported 2.22-fold higher odds of point prevalence abstinence at 26 weeks follow-up than those who did not receive such advice (10). In a systematic review of 42 trials, Stead and colleagues found that even brief advice from HCPs would likely increase quit smoking rates (11). In addition to doctors and other health professionals, tobacco cessation in oral health settings is effective and feasible (3). Yadav and colleagues found that individuals who received smoking cessation advice from a dental care professional reported 18% more quit attempts (12). In a systematic review, Holliday and colleagues found that brief advice from dental professionals increased quit rates for all tobacco use (13). Therefore, to increase tobacco use cessation rate, the United States Public Health Service (USPHS) clinical practice guidelines recommended that HCPs should use the 5A’s model (ask, advise, assess, assist, and arrange) to ask for tobacco use at every visit; advise to quit; assess willingness to quit; assist patients who are willing to make a quit attempt with counseling and pharmacotherapy; and arrange follow-up within the first week after the quit date (14). In the US, more than 80% of adults reported visiting an HCP, and more than 60% reported visiting a dentist at least once a year (15), which represents a unique opportunity for HCPs and dentists to ask about tobacco use and advise them to quit its use.

Previous studies have reported a 57.2% prevalence of medical advice to quit cigarette smoking, and sociodemographic characteristics such as Hispanic, younger age, male, and uninsured were found to be associated with a lower likelihood of receiving medical advice to quit smoking (10, 11, 16, 17). Furthermore, immigrants constitute 13.9% (18) of the US population and often experience challenges in receiving healthcare services due to cultural, social, and economic barriers (19–22). There is limited information on the prevalence of the receipt of medical advice to quit tobacco among immigrants in the US.

Although cigarette use has gradually decreased (5, 23), the emergence of a diverse landscape of combustible and noncombustible tobacco products and the observed increase in the consumption of these products (5, 8) may counterbalance the health, social, and economic benefits expected from the reduction of cigarette smoking. Therefore, it is essential to have a holistic approach in rendering medical advice to quit tobacco use. However, previous reports (10, 11, 16, 17, 24) have focused only on receiving advice to quit cigarette smoking, but not all forms of tobacco products. Furthermore, none of the previous reports included dentists as a source of medical advice to quit tobacco use (10, 11, 16, 17, 24).

To address these gaps, our study aimed to (1) investigate the prevalence of receiving advice to quit any form of tobacco products (cigarettes, e-cigarettes, cigars, cigarillos, little filtered cigars, pipes, and smokeless tobacco) from HCPs and dentists, and (2) examine disparities associated with receiving such advice among US adult tobacco users.

## 2 Methods

### 2.1 Study population and data collection

This was a cross-sectional study based on the data from NHIS 2022 (25), which collects health behavior and demographic information of US households every year. NHIS used a geographically clustered multistage sampling technique to select a sample of dwelling units. The data were collected between January 2022 and December 2022 using a computer-assisted face-to-face survey of adult household members in the non-institutionalized civilian population residing in the United States (26). Our study followed Strengthening the Reporting of Observational Studies in Epidemiology (STROBE) guidelines. Respondents to NHIS 2022 provided written informed consent, and the research ethics review board of the National Center for Health Statistics approved the survey (26). Our study was exempt from ethical approval since it was a secondary data analysis of publicly available and de-identified data.

In the NHIS 2022 survey, adults were asked questions about their visits to an HCP and tobacco use behavior in the past 12 months, which were the criteria used to determine our study population.

#### 2.1.1 HCP visit

The visit to an HCP was measured using the questions: (1) “About how long has it been since you last saw a doctor or other health professional about your health?” and (2) “About how long has it been since you last had a dental examination or cleaning?” The response options for questions (1) and (2) were “Never,” “Within past year [anytime less than 12 months ago],” “Within last two years [1 year but less than 2 years ago],” “Within the last 3 years [2 years but less than 3 years ago],” “Within the last 5 years [3 years but less than 5 years ago],” “Within the last 10 years [5 years but less than 10 years ago],” and “10 years ago or more.”. Those who responded “Within past year [anytime less than 12 months ago]” to either (1) or (2) were included in this study.

#### 2.1.2 Tobacco use

According to the NHIS report, “tobacco” refers to commercial tobacco products and not to tobacco used for medicinal and spiritual purposes by some American Indian communities (5). Thus, five tobacco products were assessed in this study: cigarettes, cigars (cigars, cigarillos, or little filtered cigars), pipes (regular pipes, water pipes, or hookahs), e-cigarettes, and smokeless tobacco. To determine tobacco use in the past 12 months, we used the following questions: (1) “Do you NOW smoke cigarettes every day, some days, or not at all?”; (2) “Do you NOW use e-cigarettes or other electronic vaping products every day, some days, or not at all?” (3) “Do you NOW smoke regular cigars, cigarillos, or little filtered cigars every day, some days, or not at all? (4) “Do you NOW smoke pipes filled with tobacco - either regular pipes, water pipes, or hookahs, every day, some days, or not at all?” (5) “Do you NOW use smokeless tobacco products every day, some days, or not at all?” The response options were “Every day,” “Some days,” and “Not at all.” Those who responded “Everyday” and “Somedays” to questions (1–5) were included in the study population. Those who responded “not at all” to question (1) were asked, “Enter time period for time since quit smoking.” The response was reported as either the number of “days,” “months,” or “years.” Those who responded >365 days, >52 weeks, >12 months, or > 1 year were excluded from the study. Therefore, our study sample consisted of 4,424 adults who reported using tobacco in the past 12 months and visiting an HCP in the past 12 months.

### 2.2 Measures

#### 2.2.1 Study outcome

The dependent variable, medical advice to quit tobacco use, was measured using the survey questions, “In the past 12 months, has a medical doctor, dentist, or other health professional ADVISED you to quit smoking or to quit using other kinds of tobacco?” The response to this question was either “yes” or “no.”

#### 2.2.2 Predictors

Selected predictors are included in Table 1. Age (recoded as <45 years/≥45 years) was chosen as a predictor because cancer risks increase after 45 due to life experiences and exposure to social and behavioral risk factors (27). Moreover, age was found to be significant in association with medical advice on quitting tobacco use in previous studies (16, 17, 24). We used sex (coded as female/male), ethnicity and race (coded as Non-Hispanic White/Non-Hispanic Black/Hispanic/Multiple/others), educational level (coded as high school or GED/less than high school/some college/bachelor’s or higher), poverty level (recoded as Below poverty level/Above poverty level), and insurance coverage (coded as covered/not covered) as predictors because they were found significant in previous studies (16, 17). The prevalence of tobacco use and quitting varies by urbanicity. Rural areas have a higher prevalence of the use of different types of tobacco products than urban areas (28, 29). However, individuals living in non-metropolitan areas were found to be less likely to receive medical advice for quitting cigarette smoking (16). Therefore, we used urbanicity (recoded as metropolitan/non-metropolitan) as a geographic predictor for our study. Furthermore, US immigrants experience challenges in healthcare service utilization due to language barriers and lack of preventive care practices (19–22). Therefore, we used place of birth (recoded as Foreign-born/US-born) as another predictor to investigate if individuals with foreign-born status experience differential outcomes in receiving medical advice to quit tobacco use.

**Table 1:** Characteristics of the adults who used tobacco products in the past 12 months in the study population stratified by the receipt of medical advice to quit tobacco use^a^ by healthcare providers- National Household Information Survey, 2022.

Characteristics	Overall		Quit tobacco use advice = Yes	Quit tobacco use advice = No
	N (weighted n)	% Weighted [95% CI^b^]	% Weighted [95% CI]	% Weighted [95% CI]
	4,424 (42385509)	100%	38.4 [36.7–40.1]	61.6 [59.9–63.3]
Ethnicity and race				
Non-Hispanic White	3,151 (29857556)	70.4 [68.5–72.3]	40.8 [38.8–42.8]	59.2 [57.2–61.2]
Non-Hispanic Black	568 (5213297)	12.3 [11.0–13.7]	38.5 [34.1–43.2]	61.5 [56.8–65.9]
Hispanic	423 (4506551)	10.6 [9.3–12.1]	25.8 [21.1–31.2]	74.2 [68.8–78.9]
Multiple/Other	282 (2808104)	6.6 [5.7–7.7]	32.6 [26.4–39.5]	67.4 [60.5–73.6]
Sex				
Female	1939 (17652264)	41.7 [40.0–43.3]	43.1 [40.5–45.7]	56.9 [54.3–59.5]
Male	2,484 (24728995)	58.3 [56.7–60.0]	35.0 [32.8–37.3]	65.0 [62.7–67.2]
Poverty				
Below poverty level	687 (5922952)	14.0 [12.8–15.3]	44.9 [40.2–49.6]	55.1 [50.4–59.8]
Above poverty level	3,737 (36462557)	86.0 [84.7–87.2]	37.3 [35.5–39.2]	62.7 [60.8–64.5]
Place of birth				
Foreign born	379 (3992763)	9.6 [8.5–10.8]	26.0 [21.4–31.1]	74.0 [68.9–78.6]
U.S born	3,962 (37619654)	90.4 [89.2–91.5]	39.9 [38.1–41.8]	60.1 [58.2–61.9]
Age (years)				
≥ 45	2,594 (21140146)	50.3 [48.4–52.3]	49.9 [47.6–52.3]	50.1 [47.7–52.4]
< 45	1811 (20848909)	49.7 [47.7–51.6]	27.1 [24.8–29.6]	72.9 [70.4–75.2]
Education				
Less than high school	415 (4338295)	10.3 [9.2–11.6]	47.2 [41.6–52.8]	52.8 [47.2–58.4]
High school/GED^c^	1,547 (15519763)	36.9 [35.2–38.6]	40.8 [37.9–43.7]	59.2 [56.3–62.1]
Some College	1,445 (13974053)	33.2 [31.6–34.9]	39.0 [36.1–42.0]	61.0 [58.0–63.9]
Bachelors or higher	992 (8251816)	19.6 [18.3–21.0]	27.8 [24.7–31.2]	72.2 [68.8–75.3]
Insurance coverage				
Covered	4,053 (38264497)	90.5 [89.3–91.6]	39.6 [37.8–41.4]	60.4 [58.6–62.2]
Not Covered	360 (4005008)	9.5 [8.4–10.7]	27.0 [21.8–33.0]	73.0 [67.0–78.2]
Urbanicity				
Metropolitan^d^	3,467 (34223825)	80.7 [78.9–82.5]	37.1 [35.2–39.0]	62.9 [61.0–64.8]
Non-Metropolitan	957 (8161684)	19.3 [17.5–21.1]	43.8 [39.8–48.0]	56.2 [52.0–60.2]

a Tobacco use assessed includes cigarettes, e-cigarettes, cigars, cigarillos, little filtered cigars, pipes, and smokeless tobacco.

b CI, confidence interval.

c GED, general equivalency diploma.

d Metropolitan includes large central metropolitan, large fringe metropolitan, medium, and small metropolitan.

### 2.3 Statistical analysis

Statistical analyses were conducted, incorporating sampling weights to account for two components of the NHIS surveys: that only a subset of the US population is selected to participate in the survey and non-response of the selected participants. The final weights are calculated in several steps: (1) each sampled individual receives a base weight, which is the inverse of the probability of being selected in the survey sample. In this initial step, households and persons within households that are more likely to be selected to participate in the survey receive lower weights. (2) Base weights are then adjusted for non-response, which can impact the representativeness of the sample. The non-response accounted weights are calibrated using iterative proportional raking, utilizing raking dimensions such as age stratified by sex, age in conjunction with race/ethnicity, levels of educational attainment, and geographical regions delineated by metropolitan statistical areas. The weighting approach ensures that the survey data is representative of the demographic distribution of the US population (30). We used weighted survey logistic regression to examine the association between medical advice to quit and several sociodemographic factors. Statistical analyses were performed using the R statistical software (Version 4.3.1), specifically utilizing the “survey” package to analyze survey data. A survey-weighted variance inflation factor greater than 10 was used as a criterion to establish any multicollinearity.

## 3 Results

**Figure 1:**
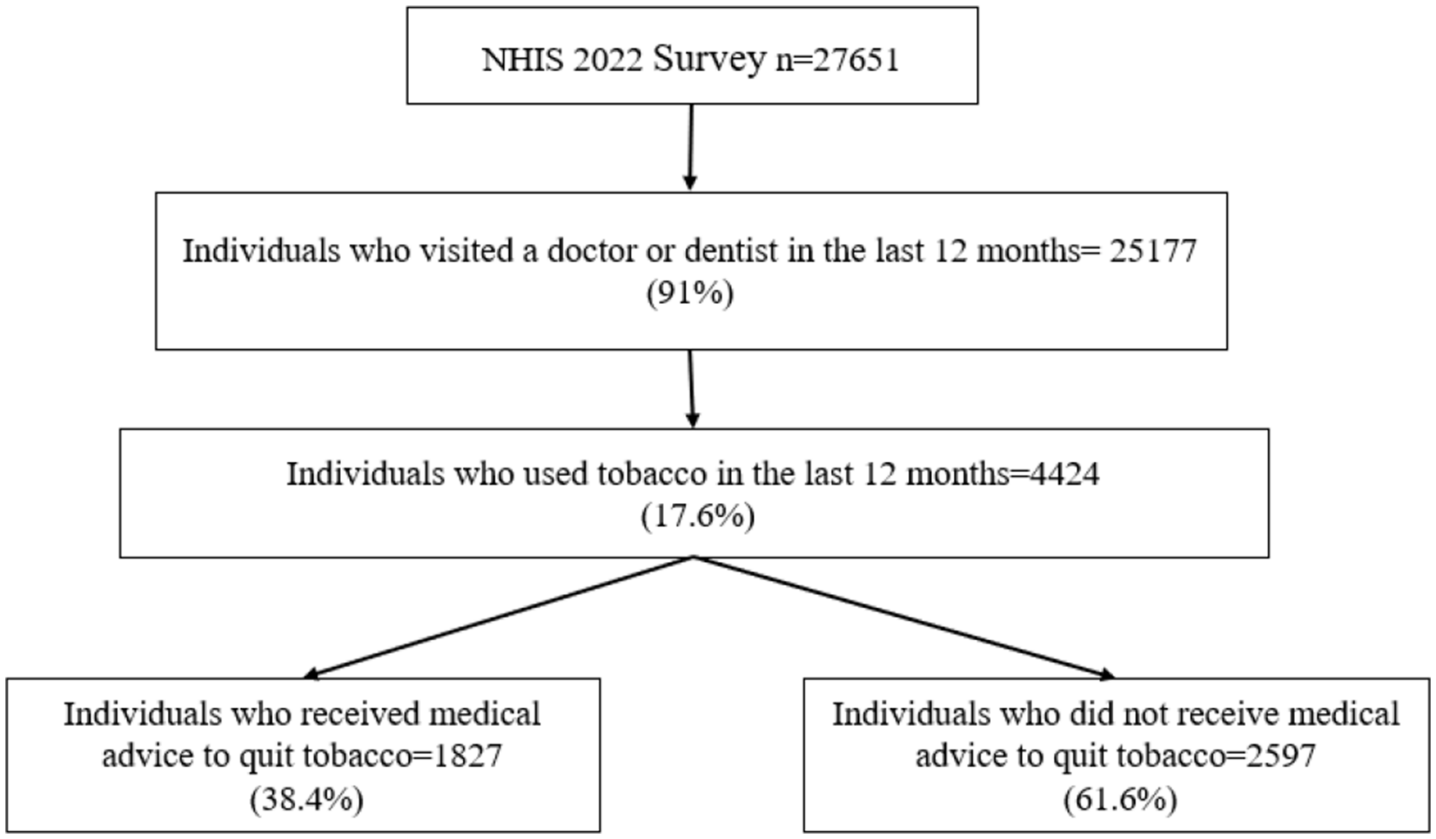
Study sample selection flowchart.

The NHIS 2022 dataset included 27,651 adult individuals, 91% of whom visited an HCP in the last 12 months. After screening for HCP visits and tobacco use in the past 12 months, the study sample included 4,424 individuals (Figure 1).

### 3.1 Characteristics of the study population

Over 70% of the study sample were Non-Hispanic White, 41.7% were females, 14% had a household income below the poverty level, 9.6% were foreign-born, 49.7% were younger than 45 years, 10.3% were individuals with less than a high school level of education, 9.5% had no health insurance coverage, and 19.3% were living in the non-metropolitan areas.

### 3.2 Prevalence of receiving HCP’s advice to quit tobacco

Overall, 38.4% (95% confidence interval [CI]: 36.7–40.1) of adult tobacco users received medical advice to quit. About 26% (95% CI: 21.1–31.2) of Hispanics, 35% (95% CI: 32.8–37.3) of males, 44.9% (95% CI: 40.2–49.6) of individuals living in households below the poverty level, 26% (95% CI: 21.4–31.1) of foreign-born, 27.1% (95% CI: 24.8–29.6) of individuals aged below 45 years, 27.8% [24.7–31.2] of individuals with education level of bachelor’s degree or higher, and 27.0% (95% CI: 21.8–33.0) of individuals without health insurance coverage received medical advice to quit tobacco use (Table 1).

### 3.3 Sociodemographic factors associated with receipt of advice to quit tobacco by HCPs

Using weighted survey logistic regression (Table 2), we found that Hispanics had 37.5% lower odds (AOR: 0.625, 95% CI: 0.464–0.843; *p* = 0.002) of receiving medical advice than Non-Hispanic White. Males had 23.3% lower odds (AOR: 0.767, 95% CI: 0.659–0.893, *p* = 0.001) of receiving medical advice to quit tobacco use than females. The odds of receiving medical advice among individuals living above the poverty level were 19.5% (AOR: 0.795, 95% CI: 0.641–0.987, *p* = 0.037) lower than those living below the poverty level. Foreign-born individuals had 33.6% lower odds (AOR: 0.664, 95% CI: 0.496–0.888, *p* = 0.006) of receiving HCPs’ advice to quit tobacco use than US-born individuals. Those younger than 45 years had 59.6% lower odds of receiving medical advice to quit tobacco use (AOR: 0.404, 95% CI: 0.344–0.473, *p* < 0.001) compared to individuals 45 years or older. Individuals with a bachelor’s degree or higher educational level had 52.3% lower odds (AOR: 0.477, 95% CI: 0.349–0.653; *p* < 0.001) of receiving medical advice to quit tobacco use than those with less than high school. The association of urbanicity and health insurance coverage with receiving medical advice for quitting was found statistically not significant.

**Table 2:** Multivariable survey-weighted logistic regression analyses correlating factors associated with medical advice to quit tobacco use^a^: National Health Information Survey, 2022.

Characteristics	AOR^b^ (95% CI^c^)	Lower 95% CI	Upper 95% CI	*P*-value
Ethnicity and race				
Non-Hispanic Black	0.85	0.68	1.06	0.14
Hispanic	0.63	0.46	0.84	0.002
Multiple/Other	0.89	0.65	1.24	0.49
Non-Hispanic White	Ref	Ref	Ref	Ref
Sex				
Male	0.77	0.66	0.89	0.001
Female	Ref	Ref	Ref	Ref
Poverty				
Above poverty level	0.80	0.64	0.99	0.037
Below poverty level	Ref	Ref	Ref	Ref
Place of birth				
Foreign born	0.66	0.50	0.89	0.006
U.S. born	Ref	Ref	Ref	Ref
Age (years)				
< 45	0.40	0.34	0.47	<0.001
≥ 45	Ref	Ref	Ref	Ref
Education				
High school or GED^d^	0.81	0.62	1.06	0.123
Some college	0.77	0.58	1.02	0.066
Bachelors or higher	0.48	0.35	0.65	<0.001
Less than high school	Ref	Ref	Ref	Ref
Insurance coverage				
Not covered	0.74	0.55	1.01	0.055
Covered	Ref	Ref	Ref	Ref
Urbanicity				
Nonmetropolitan	1.04	0.86	1.27	0.673
Metropolitan^e^	Ref	Ref	Ref	Ref

a Tobacco use assessed include cigarettes, e-cigarettes, cigars, cigarillos, little filtered cigars, smokeless tobacco, and pipes.

b AOR, Adjusted odds ratio.

c CI, confidence interval.

d GED, general equivalency diploma.

e Metropolitan includes large central metropolitan, large fringe metropolitan, medium, and small metropolitan.

## 4 Discussion

In this nationally representative sample of the US adult population who used tobacco and visited an HCP in the last 12 months, 38.4% received medical advice to quit. Although medical advice to quit tobacco use is an effective intervention for tobacco use cessation (1, 10, 11, 31), our study suggests that almost two-thirds of adult tobacco users missed clinical opportunities to quit and remain at risk of tobacco-use-related diseases. The current literature on medical advice to quit tobacco use is mainly focused on cigarette smoking. However, in practice, cigarettes and other tobacco products such as e-cigarettes, cigars, pipes, and smokeless tobacco are used solo or in combination (32). With the growing consumption of other tobacco products (33) and the rising popularity of polysubstance use (34) in the US, it is of utmost importance that every tobacco user, regardless of the type of tobacco product used, receive advice and education from their HCPs on the harms associated with tobacco use.

In our study, race and ethnicity, sex, place of birth, age, and level of educational attainment were the major sociodemographic factors found to be significantly associated with the receipt of HCPs’ advice to quit all tobacco use. We found that Hispanics were less likely to receive medical advice to quit tobacco use, which is consistent with the existing literature on tobacco smoking (19, 24, 35, 36). Among other factors, the language barrier was found contributing to the lower odds of receipt of medical advice to quit tobacco use among Hispanics (20). Therefore, culturally competent interventions should be made available to HCPs to provide advice to Hispanics to quit tobacco use (19, 37, 38).

We also found that male tobacco users were less likely to receive medical advice to quit, corroborating the existing literature (17, 35). Females are more likely to receive medical advice because they tend to seek medical assistance to quit smoking and use recommended cessation resources more frequently than males (39). Since the prevalence of tobacco use is higher among males (40), lower odds of receiving medical advice to quit in males is alarming and calls for increased efforts to encourage men to seek medical assistance to quit tobacco use.

Interestingly, foreign-born US residents were less likely to receive medical advice to quit tobacco use compared to US-born individuals. Lower odds of receiving medical advice to quit tobacco use among foreign-born could be due to physicians’ limited access to culturally competent smoking cessation interventions and patient’s lack of access to care (19, 20). Moreover, studies have reported that foreign-born individuals have lower healthcare utilization and are less likely to use preventative care (21, 22). HCPs give priority to the patient’s primary complaint and usually do not focus on smoking cessation unless they present with a smoking-related disease (41). Since foreign-born individuals are more likely to visit an HCP only when they have a health complaint, and if the complaint is not associated with any smoking-related condition, it is less likely that they will receive any quit smoking advice from the HCP (41).

We found that individuals younger than 45 years were less likely to receive medical advice to quit tobacco use, which is congruent with other studies (17, 35). According to Frank and colleagues, a physician might not find advising healthy younger patients time-efficient due to lower perceived health risk; hence, physicians focus more on older patients as they present more frequently with smoking-related diseases (42). Moreover, El-Shahawy and colleagues found that physicians are inconsistent in advising users of newer tobacco products commonly used among the youth, such as e-cigarettes, because information on the potential harms and benefits are lacking or non-consensual, and there were beliefs supporting e-cigarette use as a safer alternative to combustible products and has benefits in smoking cessation (43, 44). The 2020 Surgeon General’s report indicates that the efficacy of e-cigarettes as a smoking cessation aid is inconclusive (1), and there are health risks associated with e-cigarette use, such as nicotine dependence, cancer, respiratory infections, and oral and gastrointestinal disorders (45, 46). Under such circumstances, the lower rate of HCP advice or inconsistent advice to quit all tobacco use among younger adults reflects a missed opportunity to prevent adverse tobacco-related health issues in the future.

We also found that individuals with tertiary education (bachelor’s degree or higher) were less likely to receive advice to quit tobacco use from HCPs. The existing literature has conflicting findings on this topic. While some studies supported our findings (24, 35, 36, 47), other studies suggested that individuals with a tertiary level of education were more likely to receive medical advice to quit smoking (48–50). After adjusting for education, we found that individuals living in households above the poverty level were less likely to receive medical advice to quit tobacco use. Like education, the relationship between poverty and medical advice to quit smoking was also inconsistent in the literature. While some reports state that high-income individuals are more likely to receive medical advice (48, 50), others found no significant association between income or poverty ratio (17, 49) and medical advice. Therefore, the role of patients’ education and income on medical advice to quit tobacco use needs further investigation. A plausible explanation of our finding is that individuals from middle or high socioeconomic status (here proxied by education and poverty ratio) have a low prevalence of tobacco use (5) and have lower comorbidities (51), high literacy (52), and better living conditions, which leads to HCPs having a low perceived threat of smoking-related diseases for these patients.

Despite recommendations from the Surgeon General and other professional medical associations, medical advice to quit tobacco use remains low in the US (1). Considering the critical role of HCPs in their patient’s decision to abstain from tobacco use, the suboptimal prevalence of medical advice indicates a missed clinical opportunity to promote quitting tobacco use. This is especially concerning because, in our data, about 91% of the US adult population had a clinical encounter(s) with a healthcare professional (HCP) or a dentist in the past 12 months, indicating a missed clinical opportunity to ask for tobacco use and advice to quit to a significant proportion of the US adult population. Individual and organization-level barriers may be accountable for suboptimal medical advice to quit tobacco. At the individual level, lower odds of medical advice to quit smoking could be driven by the HCPs’ lack of perceived health risks to patients (e.g., the patient has good health), a fear of damaging patient-provider relationship (41, 42), the lack of perceived effectiveness of counseling, perceived low patient motivation, and the lack of training in tobacco use cessation and effective counseling communication (53, 54).

At the organizational level, poor reimbursement, lack of tobacco use cessation resources, limited time for patient-provider communication, and lack of support services needed for smoking cessation could account for the low receipt of medical advice to stop tobacco use (36, 55). Therefore, training HCPs on quitting tobacco use and counseling skills and developing an integrated system of HCPs advice and a tiered system of support to quit tobacco use will help improve quitting (56).

To the best of our knowledge, this is the first study that included all tobacco products (and not just cigarettes) as the primary outcome and assessed disparities in receiving medical advice to quit all tobacco use. Holistic medical advice to quit any form of tobacco is especially important now, considering the rising use of other tobacco products, such as electronic cigarettes (57) and poly use (34). Considering the established effectiveness of tobacco use cessation by oral healthcare professionals (3, 12, 13), another novelty of this study is that in addition to HCP visits, we also accounted for dental visits as a criterion for the study population, unlike previous reports (16, 19). Additionally, we included foreign-born as a predictor, which was not included in the previous studies. Finding that foreign-born individuals are less likely to receive medical advice to quit smoking gives a new insight into healthcare disparities experienced by US immigrants and should be further investigated in greater depth. Moreover, our finding on disparate medical advice to individuals with tertiary education was unique and calls for further analysis to assess intersectionality for other factors such as race and sex. Another key strength of our study is that it uses the most updated (released June 2023) nationally representative large sample of the US population.

The study had some limitations. Given the cross-sectional design, the study could not establish a causal relationship between identified factors and advice to quit tobacco use. Therefore, longitudinal studies are needed to establish the causal relationship. The data was based on self-report; hence, recall bias could have affected participants’ responses. Further, because NHIS is limited to non-institutionalized US civilian populations, results are not generalizable to institutionalized populations or persons in the military.

The prevalence of receiving HCP advice to quit tobacco use in the US adult population remains suboptimal and disparate among sociodemographic groups. Our findings call for strategic implementation of USPHS’s guidelines through policy enforcement and resource allocation to improve the receipt of medical advice to quit tobacco use among Hispanics, males, foreign-born, young individuals, and those who are highly educated. Policies should be implemented to increase training on cessation for all forms of tobacco products in medical and dental schools, as well as practitioner licensing renewal curriculums, especially for primary care physicians and dentists. More resources should be allocated to equip the HCPs with culturally adapted counseling competencies. The other targeted systemic level changes could include increasing resources and funding towards college campus health clinics and community health clinics (especially in areas with a high presence of ethnic minorities and foreign-born) to improve quit smoking.

## Data Availability

Publicly available datasets were analyzed in this study. This data can be found at: https://www.cdc.gov/nchs/nhis/2022nhis.htm.
